# Applicability of a cognitive questionnaire in the elderly and proxy

**DOI:** 10.1590/S1980-57642009DN20300012

**Published:** 2008

**Authors:** Renata Areza Fegyveres, Ana Paula Formigoni, Cláudia Sellitto Porto, Maria Teresa Carthery Goulart, Mirna Lie Hosogi Senaha, Sonia Maria Dozzi Brucki, Valéria Santoro Bahia, Paulo Caramelli, Jerusa Smid, Neuma D'Ávila Pinto Nogueira, Sandra Maria Garaude Greven, Letícia Lessa Mansur, Ricardo Nitrini

**Affiliations:** 1 Behavioral and Cognitive Neurology Unit, Division of Neurology, Hospital das Clínicas.; 2 Department of Physiotherapy, Speech-Pathology and Occupational Therapy, University of São Paulo School of Medicine.; 3 University of Minas Gerais, Vida Group.

**Keywords:** evaluation, cognition, aged, cognitive tests, screenings

## Abstract

The Informant Questionnaire on Cognitive Decline in the Elderly with the Proxy (IQCODE) was developed as a screening tool for cognition alterations.

**Objectives:**

1) To verify the applicability of IQCODE in the elderly with limited schooling,
2) To verify the reliability of the responses supplied by the aged and their proxies.

**Methods:**

Individuals of a Community Group were evaluated using the Mini-Mental State Examination (MMSE), IQCODE and Geriatric Depression Scale (GDS). The IQCODE was applied to informants and proxies.

**Results:**

We analyzed 44 individuals, aged between 58–82 years (M=66.8, SD=5.97) with mean elderly-schooling level of 3.75, SD=2.82 and 44 proxies aged 44.5 (SD=13.3), with mean schooling level of 8.25 (SD=4.3). The mean GDS was 8.22, SD=4.90 and 13 participants presented a score suggestive of depressive symptoms. The mean elderly IQCODE score was 3.26, SD=0.69 and 3.21, SD=0.65, for proxy responses. There was no statistical difference between these means. On the MMSE, the mean score was 24.20, SD=4.14 and 18 participants presented scores below the cut-off. The IQCODE answers by the elderly in this latter group were more congruent with MMSE than the answers of proxies.

**Conclusions:**

The applicability of the IQCODE in a population with little schooling was verified in that the proxy-report was similar to the elderly report. We can affirm that the elderly answers were more accurate than the proxies, as they were closer to MMSE score. The inclusion of a greater number of participants from community-dwelling settings is necessary to confirm the results obtained in this study.

## Abstract (pt)

O questionário ao informante sobre declínio cognitivo do idoso (IQCODE) foi desenvolvido como instrumento de detecção de alterações cognitivas.

**Objetivos:**

1) Verificar a aplicabilidade do questionário IQCODE a idosos com escolaridade restrita e
2) verificar a concordância de respostas entre eles e seus representantes.

**Métodos:**

Foram examinados indivíduos de um grupo comunitário com o Mini-Exame do Estado Mental (MEEM), IQCODE e Escala de Depressão Geriátrica (GDS). O IQCODE foi aplicado aos idosos e seus representantes.

**Resultados:**

Foram avaliados 44 indivíduos, com idade entre 58 a 82 anos (M=66,8, DP=5,97) escolaridade média de 3,75 (DP=2,82) e 44 representantes cuja média de idade foi de 44.5 (DP=13.3) e média de escolaridade de 8.25 (DP=4.3). A média do GDS aplicado aos idosos foi de 8,22, DP=4,90 e 13 indivíduos apresentaram escore sugestivo de sintomas depressivos. A média do IQCODE dos idosos foi de 3,26 (DP=0,69) e dos representantes 3,21 (DP=0,65). Não houve diferença estatística entre essas médias. O escore médio do MEEM foi de 24,20 (DP=4,14) e 18 participantes apresentaram pontuação abaixo da nota de corte para escolaridade. As respostas dos idosos ao IQCODE nesse último grupo foram mais congruentes com os escores do Mini-exame do estado mental do que as dos representantes.

**Conclusões:**

A aplicabilidade do IQCODE diretamente ao idoso com restrita escolaridade foi possível. As respostas dos representantes foram similares a dos idosos. Podemos admitir que as respostas dos idosos foram mais acuradas, uma vez que estavam mais próximas das notas do MEEM. A ampliação da amostra e incluindo participantes de outras comunidades é necessária para comprovação dos resultados obtidos.

One of the greatest challenges for clinicians and researchers in the cognition field is the definition of normalcy. This difficulty is accentuated when dealing with the elderly population because people age differently and this age group tends to present a greater variation in behavior. Additionally, social factors contribute to profile composition which can in turn give rise to extensive variation in performance. Such factors include lack of access to schooling or a reduced number of years of formal education. These difficulties can be minimized when, in the evaluation, the criteria for functional norms are selected through longitudinal observation and the individual’s independent and efficient performance is focused.

The Informant Questionnaire on Cognitive Decline in the Elderly (IQCODE)^1^ was developed with this purpose in mind. It is a questionnaire that assesses functionality in activities that require memory and other cognitive aspects, and is administered to the informant who is instructed to use the individual’s current performance compared with that of ten years ago as a reference.

The IQCODE presents good correlation with the Mini Mental State Examination (MMSE) and seems to be less influenced by education and pre-morbid occupation than the MMSE^2^. For this reason, it has been applied in countries with a large contingency of socially disadvantaged individuals. Furthermore, it is very reliable in the conditions of test and retest.^1,2^

In 1994, after psychometric analysis, some items on the scale were removed and, after a crossed validation study, the reduced form was found to have practically the same attributes as the extended version.^3^

The IQCODE has been translated and validated in many countries and is currently widely used on the five continents,^4^ with various different objectives: to predict the development of dementias,^5-7^ to verify the frequency of encephalic vascular accidents and correlation with dementias^8,9^ and to correlate this condition with mortality,^10^ to verify the accuracy of detection and discrimination in cases of mild cognitive impairment^11^ and the frequency of cognitive impairment prior^12^ or after^13^ encephalic vascular accidents, and the pre-existence of cognitive alterations in patients,^14,15^ to increase the accuracy of screening instruments in detecting the prevalence of cognitive and functional impairment in community dwelling subjects.^16,17^ The IQCODE is also useful in cases where it proves impossible to obtain a direct evaluation from the patient,^18^ or to compare self-report and proxy-report regarding cognitive alterations.^19^ Prior studies have demonstrated the accuracy of the IQCODE for tracking dementia^3^ to be similar to the traditional cognitive tests, with the advantage of observing changes other than cognitive loss.^5^

One Brazilian study used the IQCODE in order to evaluate the prevalence of cognitive decline in communities.^16^ Thus, it was possible to confirm the applicability in heterogeneous populations, with varying years of formal education. It was found that, although schooling had a reduced impact on the answers, there was variation in the scores of the groups composed according to the number of years of formal education. In this study, individuals with less than four years of schooling had lower scores than those with more than five years of formal education.

The IQCODE contains questions about activities that reflect transformations in modern living and could penalize the less-educated, such as referring to items that use newly developed technology, especially those which are computer-based.

Our hypothesis is that the application of the questionnaire would be feasible for the elderly (self-report), and that any differences between participant and proxy answers could be explained by socio-demographic factors.

Our objectives were to verify if the IQCODE can be applied directly to the low educated elderly and to ascertain the agreement between elderly and proxy reports on the IQCODE.

## Methods

We evaluated 44 individuals in the Vida Group, from the city of Barueri (SP) along with their proxies, both cognitively and functionally. The Vida Group is a non-governmental organization whose objective is to promote quality of life in the elderly community of Barueri. This is accomplished through a multidisciplinary team that offers assistance and education in health and social aspects. Additionally, the elderly have access to activities that focus on leisure and sports and are extended to community participation.

Cognitive screening tests and scales were applied: the Geriatric Depression Scale (GDS),^20^ the Mini-Mental State Examination (MMSE)^21^ and the IQCODE. The IQCODE was applied to proxy after the elderly gave their answers. The cut-off value for the GDS scale was 10 points or less, while for the MMSE we adopted the Brazilian reference adjusted for schooling, according to Brucki et al. (2003),^21^ and for the IQCODE the value of 3.41 was used according to Bustamante et al. (2003).^22^

Both the elderly and the proxy were evaluated individually at the Vida Group facility. The following instruction was given to the informant: *“Now, we want you to remember how your family member was 10 years ago and compare that with their state today: 10 years ago was 1996. We will ask you about situations where this person had used his memory or intelligence and we would like you to indicate if these situations have improved, remained the same, or have worsened over the past 10 years. Observe the importance of comparing their present performance with that of 10 years ago. Therefore, if 10 years ago this person had always forgotten where they put things, and they still forgets, this would be considered “not very altered”.* Each question was read aloud and then, the response options were given. The scores were presented as options that included three items, the generic (better / worse or unaltered) and the specific options, in pairs (*better / much better, worse/ much worse*). This adaptation was necessary to guarantee memorization of the instruction.

Each item was scored from 1 to 5, with 1 corresponding to the condition of “*much better*”, 2, “*better*”, 3, “*no alterations*”, 4, “*worse*”, 5 “*much worse*”, when comparing individual’s current performance to that of ten years ago.

This study was approved by the Ethics Committee of CAPEPesq n.788/02.

## Results

The mean age of the 44 elderly was 66.8, ranging from 60 to 69 years, whereas the mean age of proxy was 44.5, ranging from 30 to 44 years of age (Table 1). All proxy were elderly family members: 29 were sons or daughters and 15 were spouses of the elders, and 14 were male and 30 female.

**Table 1:** Socio-demographic data of the elderly and informants.

	Elderly M (SD) Range	Proxies M (SD) Range
Age	66.8 (5.9) 58-82	44.4 (13.3) 25-73
Schooling	3.7 (2.8) 0-12	8.2 (4.4) 0-17
GenderMF	539	1430
MMSE	24.2 (4.1) 12-30	NA
GDS	8.2 (4.9) 0-18	NA

There were illiterates in both the elderly group and proxy group, with a greater number in the former. Most of the elderly had received initial basic education. Most proxies had between 5–8 or more than 8 years of formal education.

The elderly GDS average was 8.2 (SD=4.4) and 13 scored above the cut-off (10 points). In the MMSE, the elderly mean score was 24.2 (SD=4.1) and eighteen individuals scored below the cut-off by schooling.

The mean IQCODE score was 3.21 (proxy answer) and 3.26 (elderly answer). Compared to the caregivers, a greater number of elderly subjects were complainers, although there was no statistical difference between the means. Considering the IQCODE answers provided by the elderly, 15 individuals scored above cut-off (mean 2.99, SD=0.5) compatible with impairment, six compatible with MMSE and nine false-positives, while the proxy report indicated nine cases, four compatible with MMSE and five false-positives. The difference between the IQCODE of groups with and without cognitive impairment was significant (Table 2).

**Table 2:** IQCODE scores.

	N	Elderly M (SD) Range	Proxies M (SD) Range	p
	44	3.26 (0.7) 1.31-4.69	3.21 (0.5) 1.56-4.88	0.7253
Group without cognitive impairment	26	3.15 (0.6) 1.31-4.31	3.3 (0.6) 1.75-4.78	0.3347
Group with cognitive impairment	18	3.57 (0.7) 2.06-4.69	2.99 (0.5) 1.56-3.5	0.0282

Question number 7 was reported as having the greatest loss (33 elderly and 32 proxies): *Remember where you put things that you usually keep in other places*. Participants and informants reported a similar number of complaints of worsening. Question 6 (*Remember where the things are usually kept*) also received a large number of complaints (25 elderly and proxies).

There were no significant correlations (Pearson) between GDS, MMSE, IQCODE- IQCODE-proxy although there was negative correlation between MMSE and IQCODE-elderly (r= –0.323, p=0.032).

The comparison of agreement in answers between elderly and proxies on the MMSE, IQCODE- elderly and IQCODE-proxy is presented in Table 3.

**Table 3:** Agreement between elderly and proxies*.

	_elderly_	Proxies	p
Total (44)			
MMSE × IQCODE			
No	16 (36.4%)	20 (45.5%)	
Yes	28 (63.3%)	24 (54.5%)	0.1610
IQCODE_elderly_ × IQCODE_proxy_			
No	16 (36.4%)		
Yes	28 (63.3%)		
MMSE × IQCODE_elderly_ × IQCODE_proxy_			
No		26 (59.1%)	
Yes		18 (40.9%)	
Group without cognitive impairment (32)			
MMSE × IQCODE			
No	10 (31.2%)	9 (28.1%)	0.5598
Yes	22 (62.7%)	23 (71.9%)	
IQCODE_elderly_ × IQCODE_proxy_			
No	11 (34.4%)		
Yes	21 (65.6%)		
MMSE × IQCODE_elderly_ × IQCODE_proxy_		15 (46.9%)	
No		17 (53.1%)	
Yes			
Group with cognitive impairment (12)			
MMSE × IQCODE			
No	6 (50%)	11 (91.7%)	0.7564
Yes	6 (50%)	1 (8.3%)	
IQCODE_elderly_ × IQCODE_proxy_			
No	5 (41.7%)		
Yes	7 (58.3%)		
MMSE × IQCODE_elderly_ × IQCODE_proxy_			
No		11 (91.7%)	
Yes		1 (8.3%)	

* Chi-square test.

## Discussion

The IQCODE has been widely used for cognitive-functional screening and estimating pre-morbid skills. Its contents include activities performed frequently in the daily lives of the elderly, directed towards memory and executive function.

The IQCODE was answered only by the proxy in previous Brazilian studies.^16,22^ The application directly to the elderly assumes that self-observation skills are preserved as well as the ability to recall episodes and behaviors occurring 10 years earlier. In line with previous studies, neither age nor schooling had an effect on the IQCODE score^1,2^.

The establishing of a 10-year temporal reference standard to compare previous and current cognitive conditions, helps to create a standard for the informant, however, it does not rule out all of the aspects of the informant’s referential subjectivity. In our study, we should highlight that the elderly had the opportunity to attend the Vida Group which increases valuation opportunities, social inclusion where this increased motivation might have influenced the responses of some of our participants. On the other hand, it is important to remember that the educational activities that Vida Group provides constitute an important source of motivation and cognitive stimulation. This did not prevent a large percentage of the participants (approximately one third) from reporting depressive symptoms.

We verified that the elderly tend to self attribute higher scores (*worse and much worse*) with greater frequency than the proxy.

Schooling deserves additional comments. Although Bustamante et al. found differing results according to schooling the usually considered cut-off value on the IQCODE is 3.41. We preferred to adopt this value, since it represents a safety margin to discriminate normal from compromised individuals. It is possible that this value does not represent the strict perception of less-educated individuals who made up majority of our sample.

It is supposed that the proxies with more schooling had greater expectations (higher scores on IQCODE) than the elderly regarding cognitive-functional performances of the elderly, but our study did not indicate this. There were no statistically significant differences between the global IQCODE scores assigned by the participants and the proxy. These results should be considered with the observation that there was no statistically significant correlation between MMSE and IQCODE for the elderly or proxy, although there was a slight tendency toward correlation in the former.

Specific situations of discordance in IQCODE responses between the elderly and proxies should be noted in cases of classifying the elderly into the “cognitive impairment” group. The MMSE disclosed alterations in 18 individuals. The IQCODE of these revealed congruence with MMSE in six elderly self-reports and four proxy-reports.

Considering MMSE as a gold standard, we must admit that in our sample neither the elderly nor the proxy was an ideal respondent, but the elderly perceived cognitive alterations more accurately and, possibly, earlier than the proxy. This result highlights the importance of self-report data on cognitive alterations. It should be emphasized that our elderly-participants were independent and presumably healthy. Even the group with high scores on the IQCODE (greater than 3.41) did not present severe cognitive decline.

A study with additional associated tests and larger samples could include data to properly classify these individuals as mild cognitive impairment cases and would be interesting to verify the power of cognitive screening.

Considering that there were no significant differences between the global score attributed by the elderly and proxy we could conclude that it is possible to obtain self (elderly) or proxy-reports about cognitive-functional aspects of everyday life. In cognitive screening, the reports obtained from the elderly were closer to the results of the MMSE than those of proxies.

Good applicability of the IQCODE was verified in a population with limited schooling, provided adjustments are made in the presentation. It is necessary to be cautious about extending this conclusion to a dwelling community, since our sample was engaged in activities that required a minimum of cognitive integrity.

A larger sample is necessary to confirm the results obtained in the study.
